# Prevalence of the Skipping Breakfast among the Iranian Students: A Review Article

**Published:** 2017-07

**Authors:** Mahin GHAFARI, Amin DOOSTI-IRANI, Masoud AMIRI, Zahra CHERAGHI

**Affiliations:** 1. Dept. of Public Health, School of Public Health, Shahrekord University of Medical Sciences, Shahrekord, Iran; 2. Dept. of Epidemiology, School of Public Health, Hamadan University of Medical Sciences, Hamadan, Iran; 3. Social Health Determinants Research Center, Shahrekrod University of Medical Sciences, Shahrekord, Iran

**Keywords:** Skipping breakfast, Dietary pattern, Systematic review, Iran

## Abstract

**Background:**

Adolescence is an important period since the establishment of dietary pattern can also affect the adulthood. This study aimed to estimate the overall prevalence of skipping breakfast among Iranian students.

**Methods:**

The international and national databases, including Medline, Scopus, Science Direct, Embase, Web of sciences, Google Scholar, MagIran, and SID were searched 1945–2016 as per case. All studies addressing the prevalence of skipping breakfast among Iranian students were extracted.

**Results:**

Out of 322 records, 24 articles remained for meta-analysis. The total pooled prevalence of skipping the breakfast was 0.216 (95% CI: 0.213-0.22), the girls had a higher percentage for skipping breakfast compared with boys (26% vs. 18%).

**Conclusion:**

Skipping breakfast is more prevalent in girls. Interventions are required to promote breakfast consumption in the targeted Iranian students, especially the girls.

## Introduction

In most countries, adolescents make up a large proportion of the population. According to the World Health Organization, in 2003, 19% of the world’s total populations were adolescents aged 10 to 19 yr, and 84% of them were living in the developing countries (1). In Iran, based on the results of the 2011 Population and Housing Census, the proportion of adolescents aged 10 to 19 was 16.34% (2).

Adolescence is an important period since the establishment of nutritional habits during this time can also affect adulthood (2). In addition, breakfast is known as the most important daily meal (2), and consumption of it is considered as an important indicator of a healthy lifestyle (2). Since the time between dinner and breakfast is usually the longest period without absorption of energy and nutrients, skipping breakfast may lead to metabolic changes and have a negative effect on cognitive performance (3). Eating breakfast has beneficial effects on the quality of the diet and prevents chronic disease; in contrast, skipping this meal increases risk of the metabolic syndrome and cardiovascular diseases (4, 5). So far, breakfast plays an important role in maintaining the health of children and adolescents (5).

Skipping breakfast has been reported in several countries, and skipping the breakfast meal was more common in children and adolescents (6). Some studies indicate a relation between skipping breakfast and the occurrence of obesity (7, 8). Others indicate the effect of basic characteristics (such as parent’s education and income) on skipping breakfast (9, 10).

Therefore, limited and sporadic studies have been conducted to investigate the prevalence of skipping breakfast in the Iranian student population, and the results are inconsistent. This study aimed to estimate the overall prevalence of skipping breakfast among Iranian students.

## Methods

### Searching

**Fig. 1:**
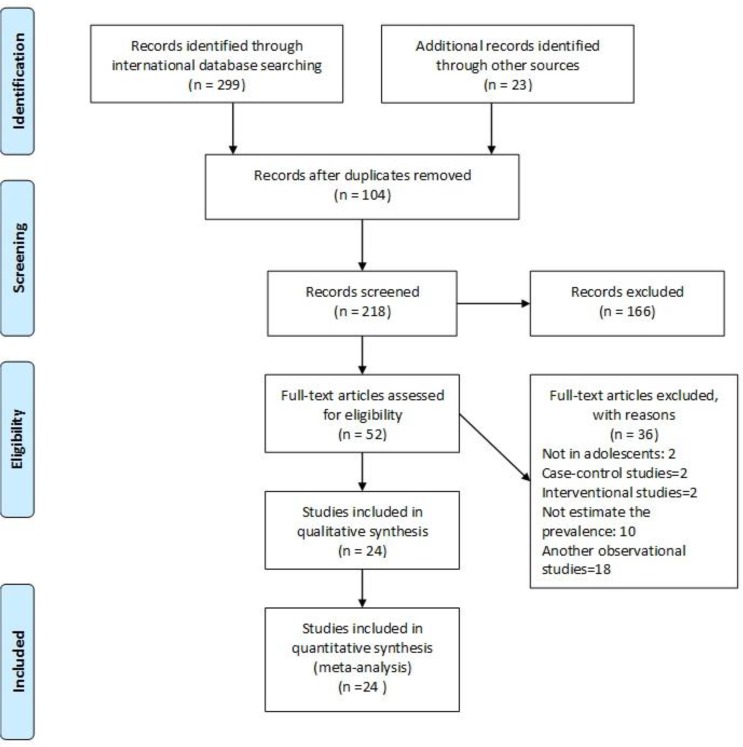
Flow chart depicting the stages of retrieving articles, checking eligibility criteria, and including the articles into the meta-analysis

Using the PRISMA guideline (11), extensive electronic and manual sources were investigated to identify relevant reviews. The international and national databases were searched using following keywords: “prevalence,” “skipping breakfast,” and “Iran.” International databases including Medline, Scopus, Embase, Web of sciences, and Google Scholar were searched up to Jun 2016. In addition, national databases including MagIran, Irandoc, Medlib, Iranmedex, and SID were searched from 1945–2016 as per case (Fig. 1).

### Inclusion criteria

All cross-sectional studies that addressed the prevalence of skipping breakfast among Iranian pupils were included irrespective of the time of the study and the language of the publication. The main outcome of interest was the prevalence of skipping breakfast.

### Data extraction and management

Two authors (M Gh. and Z Ch.) screened the titles and abstracts of the retrieved citations independently; in the next stage, they reviewed the full text of the selected studies to select the studies that met the inclusion criteria for this review. In case of missing data, we contacted the corresponding authors of the studies. The same reviewers extracted the following variables for data analysis: the year of publication, location of the study (city), mean of age, gender, education, mean of body mass index, habitat of participants, sample size, and percent of skipping breakfast.

### Assessment risk of bias

Five selected items from the Newcastle-Ottawa Scale checklist (12) were used for evaluating the quality of the included studies. The items included the following: 1) the statistical test used to analyze the data is clearly described and appropriate, and the measurement of the association is presented, including confidence intervals and the probability level (*P-*value), 2) assessment of the outcome, 3) The subjects in different outcome groups are comparable, based on the study design or analysis. Confounding factors are controlled, 4) sample size Justified and satisfactory, 5) Representativeness of the sample; truly representative of the average in the target population.

Studies that satisfied all mentioned criteria were classified as high quality. Studies that did not satisfy one item were classified as moderated quality, and studies that did not satisfy more than one item were classified as low quality.

### Assessment of heterogeneity

Statistical heterogeneity was explored using the chi-squared test at a significance level of 10%. I-square test was used to quantify the heterogeneity across the included studies (13). The variance between the studies was estimated using tau-squared statistics (14).

### Assessment of the publication bias

We used funnel plot to investigate publication bias visually (14), as well as (15)(16) tests to assess publication bias statistically.

### Estimation of summary measures

A meta-analysis was performed to estimate the percent of skipping breakfast among pupils. The inverse variance (IV) method was used for calculating the pooled estimations. Subgroup analysis was performed according to gender and the quality of the included studies. The Stata 11 (StataCorp, College Station, TX, USA) was employed for data analysis.

In order to deal with the bias caused by the size of the different populations studied (17), we used the sample size as the weight variable in the mean command in STATA.

Moreover, the meta-regression has been used to evaluate the potential factors on the heterogeneity (18). The random effect model (19) was used for data analysis and the results were reported with a 95% confidence interval.

## Results

We retrieved 322 records; 104 references were excluded because of duplication, 166 references were excluded because they were not related to the objective of the review, and 36 references were excluded because they were not eligible to be included in the meta-analysis after checking the full text. Finally, 24 articles (20–43) remained for the meta-analysis (Fig. 1 and Table 1), which included 59292 Iranian pupils, aged 7–16 yr with a mean age of 11.66 ± 0.73 yr.

**Table 1:** Characteristic of included studies in the systematic review

**Author**	**Publication Year**	**City**	**Gender**	**Education**	**Number of cases**	**Sample Size**	**Prevalence (%)**
Lotfi	2012	Zahedan	Boy	Primary	13	223	0.06
Cakirglu	2007	Tabriz	Both	Primary	12	160	0.08
Jagari	2013	Tehran	Boy	High School	6	300	0.02
Amini	2007	Tehran	Both	High School	37	389	0.10
Alimoradi	2014	Sannadaj	Both	High School	41	553	0.07
Moghadam	2011	Ghazvin	Both	Primary	136	1300	0.10
Gholami	2014	Tehran	Girl	Primary	30	164	0.18
Mortazavi	2010	Zahedan	Boy	All level	67	1278	0.05
Rashidi	2007	Tehran	Both	High School	179	2321	0.08
Djalalinia	2013	Tehran	Girl	Middle School	492	1823	0.27
Bagherniya	2014	Shahinshahr	Girl	Middle School	53	172	0.31
Shahbazi	2013	Yazd	Both	High School	36	320	0.11
Rahimi	2011	Qom	Girl	Middle School	21	100	0.21
Baygi	2015	27 province	Both	Middle & High School	227	1092	0.21
Maddah	2009	Rasht	Boy	Primary	838	3551	0.24
Hajghanai	2015	Kerman	Both	Primary	56	320	0.18
Kelishadia	2015	30 province	Both	Middle School	2627	7320	0.36
Neamati	2003	Ardabil	Girl	Middle & Primary	103	611	0.17
Ahadi	2015	31 province	Both	All Level	2548	13486	0.19
Maddah	2009	Rasht	Both	Primary & High School	2784	8937	0.31
Maddah	2009	Guilan	Girl	High School	523	2090	0.25
Veghari	2012	Golestan	Both	Primary	254	3786	0.07
Karami	2015	Omideiyeh	Male	Primary	31	155	0.20
Karimi	2008	Semnan	Both	All Level	52	1193	0.04

* Prevalence of skipping breakfast

54.05% of the studies (20 studies) referred to all five STROBE items (low risk of bias) and 18.92% (seven studies) referred to four STROBE items (moderate risk of bias). Finally, 27.3% (10 studies) referred to maximum three items (high risk of bias). The results of chi-squared and I-square tests indicate high heterogeneity for the overall prevalence of skipping breakfast. The mentioned heterogeneity test results were as follows: I-squared=99.4% and Chi squared=5953.44, *P*<0.001. Therefore, we did not report the pooled summary for the overall prevalence of skipping the breakfast.

We used the meta-regression analyses via the random effect model to investigate potential sources of heterogeneity between the studies. According to the meta-regression analysis, some factors identified as possible factors that modified the results including gender, education, and the period of conducting the study.

**Fig. 2:**
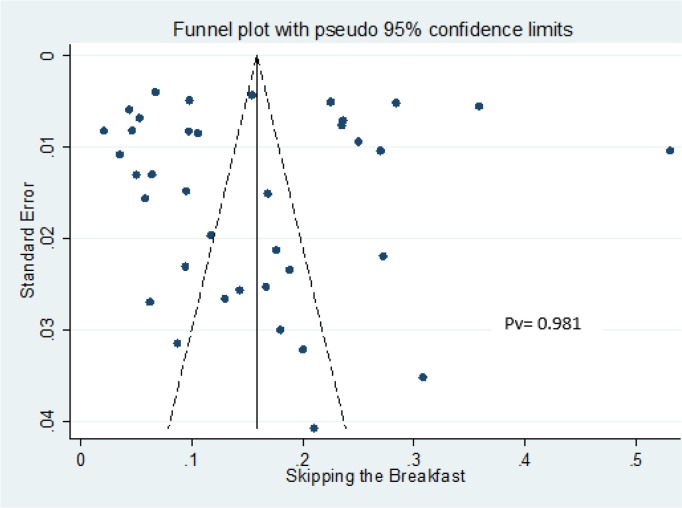
Funnel plot of included studies

We assessed the probability of publication bias using the funnel plot as well as Begg’s and Egger’s tests (Fig. 2). The studies scattered nearly symmetrically on both sides of the vertical line reflecting absence of publication bias. The results of Begg’s and Egger’s tests confirmed the absence of publication bias (*P*=0.981).

### Subgroup Analysis

We developed a subgroup analysis according to identified variables in the meta-regression analysis (gender and the quality of reporting the studies). For the variables (education and the period of the studies), the high heterogeneity remained even after subgroup analysis, and then we limited a subgroup analysis just for gender and quality of reporting the studies.

**Fig. 3:**
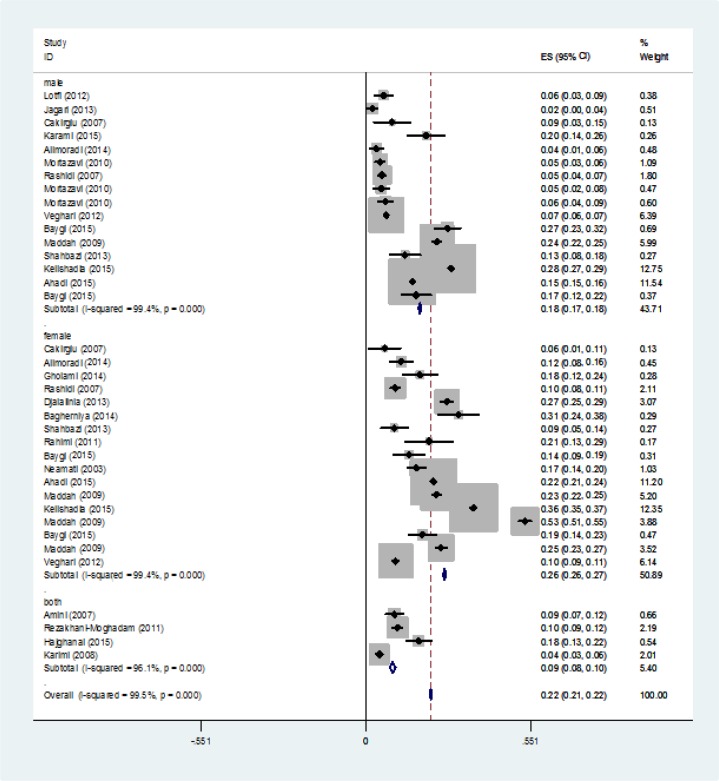
Forrest Plot of prevalence of skipping breakfast by gender

Among the high-quality studies, the girls had a higher percent of skipping the breakfast compared with the boys, but this difference was not statistically significant (23% vs. 19%, *P*=0.29). Among the moderate-quality studies, this difference was considerable and statistically significant (22% vs. 0.05%, *P* =0.02). Finally, in low-quality studies, the higher prevalence of skipping the breakfast among girls rather than boys was not statistically significant (12% vs. 0.07%, *P* = 0.25) (Table 2). Finally, the overall skipping the breakfast was 0.216 (95% CI: 0.213-0.22) (Fig. 3).

**Table 2:** Subgroup analysis of prevalence of skipping breakfast according the quality of studies and gender

**Quality of studies**	**Categories**	**n (%)**	**Pooled Prevalence**	**95% CI**	**I^2^**	*P*-value^**^
Low Risk Bias	Boy	7 (0.38)	0.19	[0.11, 0.26]	0.100	0.29
						
	Girl	11 (0.62)	0.23	[0.15, 0.30]	0.100	
						
Moderate Risk of Bias	Boy	4 (0.57)	0.05	[0.04, 0.06]	0.00	0.02
						
	Girl	3 (0.43)	0.15	[0.08, 0.36]	0.99	
						
High Risk of Bias	Boy	5 (0.62)	0.07	[0.03, 0.11]	0.88	0.25
						
	Girl	3 (0.38)	0.12	[0.06, 0.18]	0.77	

** *P*-value of Subgroup Difference

## Discussion

In this systematic review, we retrieved 24 studies that addressed the prevalence of skipping breakfast in Iranian pupils aged 7–16 yr. Because of the high heterogeneous results, we conducted a subgroup analysis based on the meta-regression analysis via random effect model. In the meta-regression analysis, some factors had potential effects on the heterogeneity such as gender, education, period of conducting the studies, and quality of studies.

Based on the subgroup analysis, in all levels of quality (high, moderate, and low), the prevalence of skipping breakfast among girls was more than boys were. However, the mentioned difference was statistically significant only in the moderate quality group. In addition, some primary studies that conducted in other countries also indicated greater prevalence of skipping the breakfast among girls in comparison to boys (11, 44, 45). According to the result of the researchers at Edinburg University, because of eating habits (e.g., Consumption of daily sugary drinks and snack type foods, such as sweets and crisps), girls skipped breakfast more often than boys (46).

Poor socioeconomic status was strongly associated to skipping breakfast in the Iranian population (4, 47, 48). In addition, eating breakfast was more common in pupils more educated parents and acceptable economic status. In this systematic review, we could not assess the prevalence of skipping breakfast by socio-economic status since the majority of the studies did not assess the socio-economic status of pupils.

One of the most important limitations of this study was that we did not assess the other important factors that affect breakfast skipping, such as socio-economic, behavioral (hours of sleeping at night, time of waking up in the morning, and level of physical activity), environmental (influence of friends and parents), and cognitive factors (perceived barriers, self-efficacy, and attitudes). Additionally, most studies did not report the mentioned information, while several observational studies showed some evidence of mentioned factors on skipping breakfast in different populations (3, 44, 49).

## Conclusion

Skipping breakfast is more prevalent in girls than boys are. This study again highlighted the interventions required to promote breakfast consumption in the targeted Iranian students, especially in girls.

## Ethical considerations

Ethical issues (Including plagiarism, informed consent, misconduct, data fabrication and/or falsification, double publication and/or submission, redundancy, etc.) have been completely observed by the authors.
